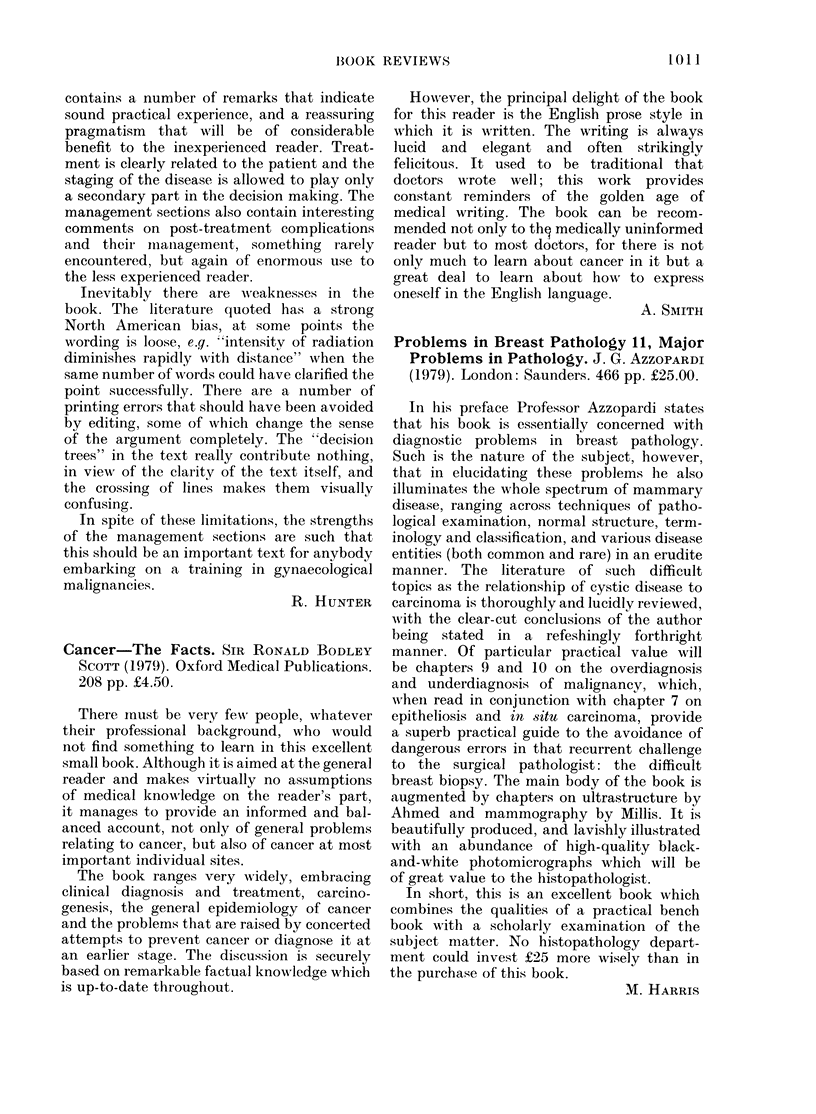# Problems in Breast Pathology 11, Major Problems in Pathology

**Published:** 1980-06

**Authors:** M. Harris


**Problems in Breast Pathology 11, Major Problems in Pathology.** J. G. AZZOPARDI (1979). London: Saunders. 466 pp. £25.00.

In his preface Professor Azzopardi states that his book is essentially concerned with diagnostic problems in breast pathology. Such is the nature of the subject, however, that in elucidating these problems he also illuminates the whole spectrum of mammary disease, ranging across techniques of pathological examination, normal structure, terminology and classification, and various disease entities (both common and rare) in an erudite manner. The literature of such difficult topics as the relationship of cystic disease to carcinoma is thoroughly and lucidly reviewed, with the clear-cut conclusions of the author being stated in a refeshingly forthright manner. Of particular practical value will be chapters 9 and 10 on the overdiagnosis and underdiagnosis of malignancy, which, when read in conjunction with chapter 7 on epitheliosis and *in situ* carcinoma, provide a superb practical guide to the avoidance of dangerous errors in that recurrent challenge to the surgical pathologist: the difficult breast biopsy. The main body of the book is augmented by chapters on ultrastructure by Ahmed and mammography by Millis. It is beautifully produced, and lavishly illustrated with an abundance of high-quality black-and-white photomicrographs which will be of great value to the histopathologist.

In short, this is an excellent book which combines the qualities of a practical bench book with a scholarly examination of the subject matter. No histopathology department could invest £25 more wisely than in the purchase of this book.

M. HARRIS